# Immune evasion in acute myeloid leukemia: current concepts and future directions

**DOI:** 10.1186/2051-1426-1-13

**Published:** 2013-08-27

**Authors:** Ryan M Teague, Justin Kline

**Affiliations:** 1 Department of Molecular Microbiology and Immunology, Saint Louis University School of Medicine, 1100 S. Grand Blvd, St. Louis, MO 63104, USA; 2 Department of Medicine, University of Chicago Comprehensive Cancer Center and Committee on Immunology, 5841 S. Maryland Ave, Chicago, IL, MC 2115, 60637, USA; 3 University of Chicago Comprehensive Cancer Center and Committee on Immunology, Chicago, IL, USA

**Keywords:** Acute myeloid leukemia, Immune evasion, Anti-tumor immunity

## Abstract

Immune responses generated against malignant cells have the potential to inhibit tumor growth, or even eliminate transformed cells before a tumor forms. However, immune tolerance mechanisms that normally protect healthy tissues from autoimmune damage pose a formidable barrier to the development of effective anti-tumor immunity. Because malignant cells are derived from self-tissues, the majority of defined tumor antigens are either shared or aberrantly expressed self-proteins. Eliciting productive T cell responses against such proteins is challenging, as most high-affinity, self-reactive T cells are purged during thymic selection. Some T cells capable of tumor antigen recognition escape thymic deletion, but are functionally inhibited by peripheral tolerance mechanisms which limit their ability to attack a developing malignancy. Alternatively, some tumors express antigens derived from mutated self-proteins, viral proteins or self proteins expressed only during embryonic development. These antigens are recognized by the immune system as foreign and could be recognized by a relatively large number of peripheral T cells. Even in this scenario, tumors evade otherwise effective T cell responses by employing potent immunosuppressive mechanisms within their local environment. In the setting for solid malignancies, such as melanoma, a growing number of putative immune evasion mechanisms have been characterized. However, acute myeloid leukemia (AML) is a systemic disease, and the pathways it exploits to subvert the host immune response may be quite different than those of a solid tumor. Much remains unknown regarding the immune escape mechanisms promoted by AML, and whether efforts to thwart tolerance may influence the progression of this disease. Here, we review current concepts of immune evasion in AML, and speculate how potentially effective immunotherapeutic strategies might be developed to reverse immune tolerance in leukemia patients in the future.

## Introduction

Acute myeloid leukemia (AML) is the most common acute leukemia in adults. Treatment with modern chemotherapy regimens often induces complete remission, but a majority of patients will ultimately relapse and succumb to the disease. For many years it has been recognized that allogeneic stem cell transplantation can be curative for some patients with AML [1]. The major therapeutic effect of allogeneic stem cell transplantation is derived from the so-called graft-versus-leukemia effect thought to result from recognition of minor histocompatibility antigens expressed on malignant cells in the host by donor-derived T cells [2-5]. The existence of a graft-versus-leukemia effect has been further supported by evidence that infusions of donor lymphocytes can induce disease remission in patients who have relapsed following allogeneic stem cell transplantation [6]. Unfortunately, only a minority of patients with AML are candidates for this procedure. Therefore, cancer immunologists have sought approaches to stimulate anti-leukemia immunity within the host to promote immune-mediated elimination of AML.

In order to exploit the immune system to eradicate leukemia cells, it is imperative to identify leukemia-specific antigens that could be targeted, for example, through vaccination or adoptive cell therapy. Over the past decade, a growing number of AML peptide antigens have been discovered, including those derived from Wilm’s tumor protein 1 (WT1), Proteinase 3 (PR3), receptor for hyaluronan acid-mediated motility (RHAMM), and others [7]. Many of these antigens are over-expressed, but not uniquely expressed by AML cells, and are thus classified as tumor-associated antigens (TAA). Tumor-specific antigens (TSA) uniquely expressed by AML cells have not been characterized. Although AML cells express antigens recognizable to host T cells, established leukemia is rarely eradicated by the host immune system. One contributing factor is that AML-derived antigens are also expressed in other tissues, including the thymus. Thus, T cells in the periphery capable of recognizing these antigens do so with low avidity and are only able to elicit a weak immune response upon encountering antigen-expressing AML cells *in vivo*. Secondly, malignancies including AML employ a number of immune evasion mechanisms which inhibit the generation or functional execution of anti-tumor immune responses. Over the past 15 years, a number of putative tumor escape mechanisms have been identified, largely in the setting of solid malignancies such as melanoma. They include: establishment of a metabolically hostile microenvironment [8,9], poor T cell costimulation by tumor cells leading to T cell anergy [10], expression of negative costimulatory ligands such as PD-L1 and Gal-9 within the tumor environment [11,12], production of immune suppressive cytokines and enzymes [13,14], expansion and/or induction of suppressive cell types (regulatory T cells (Tregs), myeloid-derived suppressor cells (MDSC) and macrophages) [15,16]. Many of these pathways are activated in concert in the cancer-bearing host, thereby generating a network of interactions which are inhibitory to T cell function and provide a permissive environment for tumor progression.

The local environment created by AML cells to promote their survival and prevent their rejection by the host immune system has been relatively under-explored. Because AML is a systemic disease at its inception, it seems reasonable to speculate that the mechanisms which regulate immune activation versus tolerance in the leukemia-bearing host may be quite different than those in a host with a solid malignancy. In this review, we will focus on what is currently known about immune evasion in AML, considering data from pre-clinical models, as well as from AML patients. Additionally, we will speculate how these pathways may be interfered with to enhance the efficacy of immunotherapeutic approaches for AML patients in the future.

## Review

### Putative mechanisms of cancer immune evasion

Tumor infiltrating lymphocytes (TIL) are present in many human cancers. Among TIL are tumor antigen-specific T cells which should theoretically be able to recognize and eliminate antigen-expressing tumor cells. However, even when heavily infiltrated by immune cells, malignancies often continue to progress, suggesting that the tumor environment actively inhibits the execution of anti-tumor immune responses. In rapidly progressing cancers, the by-products of metabolism can impair T cell function indirectly by generating hypoxic and acidic conditions [8,9]. A number of additional immune evasion mechanisms exploited by tumors are specifically tailored to subvert T cell immunity, and are discussed here with a focus on their role in promoting immune escape by AML.

### Circumventing immune surveillance

CD8^+^ and CD4^+^ T cells continuously survey antigenic peptides presented by major histocompatibility complex class I (MHC-I) and MHC-II molecules, respectively. Lost or reduced expression of MHC molecules by cancer cells has long been appreciated as a major impediment to the ability of T cells to “see” and subsequently eliminate malignant cells [17]. Diminished MHC expression on malignant cells may occur due to mutations in the cellular machinery necessary to present peptide/MHC on the cell surface [18], or because of immunoediting by T cells that selectively kill tumor cells expressing higher concentrations of antigen/MHC-I and leave behind those not so easily recognized [19]. Outgrowth of these antigen-loss variants is much harder to contain by immunological means [20,21]. Loss of antigen/MHC-I has been described in a wide range of human cancers [18], including relapsing AML [20,21]. For example, Vago et al. studied 17 AML patients who relapsed following haploidentical stem cell transplantation and infusion of donor-derived T cells. AML cells from 5 of these patients had lost expression of the HLA haplotype which differed from that of the donor-derived cells through the process of uniparental disomy of chromosome 6p. Donor T cells were unable to recognize and kill AML cells procured at the point of relapse because they no longer expressed the restricting HLA molecules required for T cell recognition. This fascinating study provided evidence that AML cells can evade the host immune response through immunoediting; a potentially critical barrier to maintaining the graft-versus-leukemia response following allogeneic stem cell transplantation, and possibly following other immune-based therapies.

### Exploiting negative regulatory receptors

Negative regulatory receptors on T cells initiate intracellular signaling events that interrupt activation cascades following antigen recognition and costimulation. This naturally limits T cell-mediated damage to self-tissues by modulating T cell responses once invading pathogens are cleared, but also presents a major obstacle to eliciting durable anti-tumor immunity. For example, tumor cells can engage these pathways within T cells by up-regulating inhibitory ligands [22,23]. Intriguingly, enhanced expression of inhibitory ligands by tumor cells results from local IFNγ secretion by activated TIL, revealing a remarkable adaptation by which tumors can hijack an otherwise protective immune response. Thus, it is not surprising that several regulatory receptors have become targets for cancer immunotherapy.

The most extensively studied negative regulatory receptors are members of the CD28 family, and include cytotoxic T lymphocyte antigen-4 (CTLA-4) and programmed cell death-1 (PD-1), which bind distinct members of the B7 family of ligands. CTLA-4 is expressed on activated T cells, as well as regulatory T cells, and binds to the ligands B7.1 (CD80) and B7.2 (CD86) on antigen presenting cells (APC). CTLA-4-deficient mice develop a rapidly lethal T cell-mediated autoimmunity [24], which clearly demonstrates the importance of CTLA-4 in down-regulating T cell activation. On effector T cells, CTLA-4 has a much higher affinity for B7 molecules, and is able to “outcompete” CD28 for access to the immune synapse, thereby limiting costimulation. Furthermore, CTLA-4 engagement of B7 ligands diminishes APC function by modulating B7 expression and function in these cells. [25]. Blockade of CTLA-4 enhances tumor immunity in animal models of cancer, which is thought to rely on the enhancement of effector T cell activity, and the inhibition or deletion of regulatory T cells [26-28]. The potential of CTLA-4 blockade to mobilize anti-tumor immunity has been recapitulated in clinical trials for human melanoma and other solid tumors [29-31], and in 2011 a monoclonal anti-CTLA-4 antibody (Ipilimumab) received FDA approval for the treatment of patients with advanced melanoma.

Data supporting a significant negative regulatory role of CTLA-4 in AML are limited. LaBelle et al. demonstrated in a pre-clinical AML model that CTLA-4 blockade had no significant impact on disease progression unless leukemia cells were engineered to express B7 molecules [32]. On the other hand, CTLA-4 blockade resulted in a significantly enhanced expansion of functional human AML-specific T cells cultured with autologous AML cells [33]. Based on the favorable experience of CTLA-4 blockade in human solid tumors, it is now being evaluated in clinical trials of patients with AML in relapse (NCT01757639) or following allogeneic stem cell transplantation (NCT01822509; NCT00060372).

PD-1 (CD279) is a receptor expressed on the surface of activated T cells, B cells, and NK cells which binds to either its broadly expressed tissue ligand PD-L1 (B7-H1/CD274) or to its hematopoietically-restricted ligand PD-L2 (B7-DC/CD273) [34,35]. PD-1 is commonly expressed on T cells in the solid tumor environment, and a wide variety of malignant cells (including AML cells) express PD-L1. The expression of PD-1 on T cells and PD-L1 on malignant cells impacts negatively on clinical outcome in several cancer subtypes [11,36,37]. These early observations provided strong rationale for targeted blockade of PD-1/PD-L1 interactions first in pre-clinical cancer models and ultimately in humans with cancer.

As with CTLA-4, PD-1-deficient mice also develop autoimmunity, but the disease is less severe and occurs later in life [38]. The cytoplasmic tail of PD-1 contains a signaling motif that when phosphorylated during ligation recruits SHP phosphatases and disrupts CD28 and TCR signaling cascades, specifically targeting the PI3K and Akt pathways [39]. Studies in animal models have convincingly shown that blockade of the PD-1 pathway can boost T cell immune responses in solid tumors as well as in AML. Zhang and colleagues demonstrated that leukemia antigen-specific T cell responses and survival following AML induction were significantly superior in PD-1-deficient mice, and also in wildtype mice following administration of anti-PD-L1 blocking antibodies [40]. Likewise, PD-1 and PD-L1 blockade have produced exciting results in patients with a range of different solid cancers [41,42], and PD-1 blockade is now being investigated clinically in AML patients in combination with a dendritic cell-based cancer vaccine (NCT01096602). It is also interesting to speculate that the use of checkpoint blocking antibodies to stimulate a graft-versus-leukemia effect following allogeneic stem cell transplantation may be an efficacious strategy. However, due to the non-antigen-specific T cell stimulatory capability of these agents, it is also possible that they may also promote graft-versus-host disease. Currently, there are several studies of CTLA-4 blockade in patients who have either relapsed or who have persistent leukemia following allogeneic stem cell transplant (NCT00060372, NCT01822509), though to our knowledge, PD-1 blockade in the post-transplant setting has not yet been tested in humans.

Additional non-CD28/B7 family receptors such as lymphocyte-activation gene-3 (LAG-3) and T cell immunoglobulin domain and mucin domain 3 (TIM-3) have been identified as potential targets in cancer [43]. LAG-3 is a CD4-like molecule that binds to MHC-II with high affinity, and is primarily expressed on NK cells, γδ T cells, Tregs and activated αβ T cells [44,45]. The mechanisms by which LAG-3 regulates immune responses are not yet completely understood. When expressed on activated or exhausted T cells, LAG-3 acts as an inhibitory receptor that requires the activity of a KIEELE motif in the cytoplasmic tail [46]. Preclinical studies have shown that disruption of the LAG-3 pathway can be achieved by antibody blockade or genetic ablation to restore T cell responses against tumors. LAG-3 blockade is especially potent when combined with PD-1 pathway inhibition [47-51]. However, LAG-3 is also expressed on Tregs, where it engages MHC-II on APC and inhibits their ability to elicit effector T cell responses [52].

Clinically, LAG-3 blockade has been exploited by administering a soluble form of the receptor (IMP321) to patients with viral infections (NCT00354263, NCT00354861) or those with different cancers including melanoma, breast cancer, renal cell carcinoma, and pancreatic cancer (NCT01308294, NCT00349934, NCT00351949, NCT00732082), but has not been tested in AML patients to our knowledge. This clinical-grade soluble LAG-3 is reported to target and activate a subset of dendritic cells through ligation of MHC-II [53,54], which seems contradictory to the role of LAG-3 on Tregs. It is possible that IMP321 directly influences APC, Tregs and effector T cells simultaneously to enhance immunity, but this has yet to be demonstrated experimentally.

TIM-3 is an inducible negative regulatory receptor on T cells that binds the ligand Galectin-9 [55]. Identified early on as a marker of dysfunctional T cells in HIV patients [56], TIM-3 expression has more recently been observed on dysfunctional T cells within the solid tumor environment in pre-clinical models and from primary human melanomas [12,57]. Combined TIM-3 and PD-1 expression on TIL identifies a particularly “exhausted” T cell population [12,57]. TIM-3 blocking antibodies result in enhanced anti-tumor T cell responses and control of tumor growth in murine solid cancer models, especially when combined with PD-1 and CTLA-4-blocking antibodies [12,58,59]. In pre-clinical AML models, TIM-3-expressing T cells demonstrated a reduced ability to produce effector cytokines, and typically co-expressed PD-1 [60]. Blockade of TIM-3 yielded a modest effect on survival following AML induction, while combined TIM-3 and PD-L1 blockade demonstrated a synergistic effect [60]. Collectively, these data suggest that TIM-3 blockade may also be effective in re-awakening T cell responses in cancer patients, including those with AML, particularly in combination with anti-PD-1 or anti-CTLA-4 therapy.

Initial clinical trials testing the efficacy of so-called “checkpoint blockade” with single agents, such as CLTA-4, PD-1 or PD-L1 blocking antibodies have clearly demonstrated their potential to augment immune responses against a range of human cancers, but have also revealed a limited benefit of monotherapy for the majority of treated patients [29,41,42,61]. An emerging strategy to further improve anti-tumor immunity in cancer patients is through combinatorial blockade of negative regulatory receptors on T cells. This approach is supported by results from experiments in pre-clinical cancer models (including AML models), in which combined checkpoint blockade was synergistic with regard to promoting enhanced anti-tumor T cell responses and also improved control of tumor progression [12,47,49,60,62]. This effect is based on the idea that simultaneous blockade of several non-redundant negative regulatory pathways is likely to maximally re-engage multiple T cell functions for optimal anti-tumor immunity. For example, PD-1 and CTLA-4 blockade was recently shown to prevent the deletion of tumor-reactive T cells, whereas additional blockade of LAG-3 was vital to promote greater effector function among these persisting TIL – providing a clear survival benefit for leukemia-bearing hosts [47]. Several clinical trials are now underway to evaluate safety and efficacy of combined PD-1 and CTLA-4 blockade in patients with renal cell carcinoma, non-small cell lung cancer, or melanoma (NCT01783938, NCT01472081, NCT01454102, NCT01024231). However, in light of the potentially life-threatening autoimmune complications which can occur with single-agent CTLA-4, PD-1 and PD-L1 antibody blockade, [29,31,41,42,63], caution will be needed when combining these agents. It is tempting to speculate that optimal combinations may allow for lower doses of individual antibodies, thereby reducing potential toxicity to healthy tissues while maintaining robust anti-tumor immunity. Indeed, one human trial has recently reported that concurrent blockade of CTLA-4 and PD-1 was synergistic in generating anti-tumor responses in melanoma patients, while adverse immune-related toxicities were no more prevalent than with monotherapy [64]. There are presently no clinical trials to our knowledge evaluating combined checkpoint blockade in AML patients.

### Accumulating suppressor cell populations

Regulatory T cells (Tregs) are a naturally immunosuppressive CD4^+^ T cell population defined by their expression of the FoxP3 transcription factor, which is necessary for their developmental and functional programs [65,66]. Loss of function mutations in the *FoxP3* locus result in severe autoimmune complications in mice and humans, highlighting the critical role of Tregs in the maintenance of peripheral tolerance to self-antigens [67,68]. Tregs can develop in the thymus as “natural” Tregs, or be induced in the periphery from conventional CD4^+^ T cells following exposure to TGF-β or retinoic acid [69]. Treg-mediated suppression of effector T cells is accomplished through diverse mechanisms that include competition for IL-2 [70], secretion of inhibitory cytokines, such as IL-10, IL-35 [71], and TGFβ [72], production of extra-cellular adenosine [73], and by altering the function of antigen presenting cells through their engagement of CTLA-4 and LAG-3 expressed on Tregs [52,74,75].

Tregs accumulate in the peripheral blood and within tumors in a wide variety of malignancies. In many cases, the density of tumor-associated Tregs correlates directly with disease stage (i.e. tumor burden) [16]. Dozens of studies have attempted to link the Treg frequency in the tumor environment with clinical outcome [76], with varying results. However, a majority have indicated that a high Treg density in the tumor tends to correlate unfavorably with clinical outcome [77]. Whether Tregs play a causal role in human cancer development or progression remains to be proven.

Reports of Treg accumulation in cancer-bearing patients inspired a host of pre-clinical studies to determine whether Treg depletion would enhance anti-tumor immune responses. In fact, inhibition or depletion of Tregs in a number of transplantable cancer models resulted in significantly enhanced anti-tumor T cell responses and control of tumor progression [78-80]. In an attempt to translate these exciting pre-clinical observations into cancer patients, and taking advantage of the constitutive expression of the IL-2 receptor α-chain by the majority of Tregs, several groups have tested whether a fusion protein of IL-2 and diphtheria toxin (denileukin diftitox) could deplete Tregs in this setting. The results from these experiments have been mixed. An early report from Dannull and colleagues concluded that denileukin diftitox significantly reduced Treg numbers in patients with metastatic renal cell carcinoma which led to enhanced T cell responses following vaccination [81]. Morse et al. also found that denileukin diftitox depleted Tregs in cancer patients, but only following multiple administrations. Here again, Treg depletion correlated with enhanced T cell responses raised following vaccination [82]. However, multiple studies have failed to repeat the early promising results with this agent, and reported either no or a very transient reduction in circulating Tregs following its administration [83,84], which has tempered enthusiasm for this strategy. More recently, effective and somewhat durable Treg depletion has been demonstrated in cancer patients following administration of anti-CD25 monoclonal antibodies (Daclizumab) [85-87]. Treg depletion led to enhanced adaptive immune responses raised following vaccination in 2 of 3 studies [86,87], while its administration appeared to diminish functional antigen-specific T cell responses in another study, possibly because the anti-CD25 antibody also depleted activated T cells which can also express CD25 [85]. Overall, these studies suggest that Treg depletion is achievable through anti-CD25 antibody administration, but also that dose and schedule may be critical factors to consider.

In AML, several groups have observed elevated Treg frequencies in the peripheral blood and bone marrow of AML patients compared to controls [88-90]. In these retrospective studies, Treg frequency in the peripheral blood of AML patients appeared to correlate negatively with response to induction chemotherapy and survival. Tregs from AML patients are also more suppressive in vitro than those from healthy controls [89]. In murine AML models, Tregs accumulate in leukemia-bearing mice [[91] and J. Kline unpublished observations], and their depletion alone or in combination with PD-L1 blockade resulted in enhanced anti-leukemia T cell responses [91,92]. For these reasons Treg ablation or inhibition remain attractive strategies to promote effective anti-tumor immunity in humans with AML. Clinical studies are now underway to investigate if T cell responses to tumor vaccines are augmented in AML patients following depletion of Tregs (NCT01513109; NCT01842139).

Over 25 years ago, populations of suppressive myeloid cells were found to expand in cancer-bearing animals [93]. These immature myeloid cells, now called myeloid-derived suppressor cells (MDSC), are comprised of a heterogeneous mixture of cells morphologically resembling either monocytes or granulocytes [94]. In mice, MDSC are characterized by the expression of CD11b and Gr-1 [95], while in humans MDSC are described as CD11b^+^CD14^-^CD33^+^ cells [96]. Present at very low frequencies outside of the bone marrow in healthy individuals, MDSC are capable of massive expansion in cancer patients, where they potently inhibit the proliferation and effector function of anti-tumor T cells through their production of arginase, nitric oxide and reactive oxygen species [15]. MDSC can also promote Treg induction [97]. In pre-clinical cancer models, effective strategies have been employed to either promote MDSC maturation [98], modulate their function [99], or target them for depletion [100,101]. Therapeutic targeting of MDSC in humans with cancer has not been successfully accomplished to date. MDSC also expand in animal AML models [102], but their role in suppressing the proliferation and function of leukemia antigen-specific T cells has not been explored in mice or humans.

### Inhibitory enzymes

Indolamine 2,3-dioxygenase (IDO) is an enzyme which catalyzes the rate-limiting step in tryptophan degradation along the kynurenine pathway [103]. T cells are extremely sensitive to environmental levels of tryptophan, and its local depletion strongly inhibits T cell proliferation, while tryptophan metabolites negatively regulate T cell activation and survival [104]. IDO is expressed by hematopoietic cells, such as plasmacytoid dendritic cells and immature DC [105], and also by non-hematopoietic cells, including bone marrow-derived mesenchymal cells [106]. A seminal study implicated IDO in cancer immune evasion after its expression was demonstrated in a wide variety of human malignancies [14]. In a murine model, IDO-expressing tumor cells grew progressively, while those negative for IDO expression were rejected. Numbers of tumor antigen-specific T cells were significantly lower in mice with IDO-expressing tumors, suggesting that IDO negatively regulated T cell proliferation. Following administration of 1-methyltryptophan (1-MT), a competitive inhibitor of IDO [107], IDO-expressing tumors progressed much more slowly, and this effect was T cell-dependent [14].

IDO expression has also been described in AML. Curti et al. observed IDO expression among half (40 of 76) of AML bone marrow samples tested [108]. Similar to many solid tumors, IDO expression by AML cells appeared to be constitutive, and was not upregulated by IFN-γ, arguing that its expression by AML cells might occur as part of the transformation process, supported by the finding that normal CD34^+^ hematopoietic precursor cells did not express the IDO protein. The enzymatic activity of IDO is also enhanced in the blood of AML patients compared to controls [109].

Expression of IDO by plasmacytoid dendritic cells in tumor-draining lymph nodes negatively regulates T cell function by activating suppressive pathways within mature Tregs [110,111]. In a fascinating set of experiments, Curti et al. identified increased circulating Treg frequencies in leukemia patients whose AML cells expressed IDO [112]. IDO-expressing AML cells were able to induce FoxP3 expression in conventional CD4^+^CD25^-^ T cells which developed suppressive capabilities *in vitro*. IDO inhibition through 1-MT completely abrogated this effect. Collectively, these observations support a model in which IDO production, either by dendritic cells or by AML cells promotes immune evasion by directly inhibiting proliferation of tumor-specific T cells, or indirectly by promoting Treg induction and/or suppressive capability.

Based on these data, and because IDO expression by AML cells may result in poor clinical outcomes [109,113], inhibition of IDO may be a promising approach to combat immune evasion by AML. Recently, extremely potent IDO inhibitors, such as INCB024360 (Incyte Corporation), have been developed and appear to be highly active in pre-clinical cancer models [114]. Several early-phase studies evaluating the efficacy of INCB024360 are ongoing in solid malignancies (NCT01685255; NCT01604889), and in myelodysplastic syndrome (NCT01822691), a disease that often progresses to AML.

### Deletional T cell tolerance

Our laboratory has recently uncovered a previously undescribed mechanism of peripheral tolerance which occurs quite rapidly in hosts harboring AML [102]. Using the C1498 AML model, we explored leukemia-antigen specific T cell responses to AML cells inoculated either subcutaneously (to mimic a solid tumor) or intravenously (to model a leukemia). While functional T cell responses against subcutaneously-inoculated AML cells were vigorous, those generated following intravenous AML cell inoculation were quite poor. Interestingly, an intravenous challenge with AML cells prevented T cell activation following a subsequent subcutaneous AML cell challenge in the same animal, suggesting that the systemic presence of AML cells actively promoted peripheral T cell tolerance. T cell receptor transgenic T cells specific for our model tumor antigen underwent abortive proliferation in hosts following a systemic AML cell challenge. Over-expression of the anti-apoptotic Bcl-XL protein in leukemia antigen-specific T cells completely restored their ability to accumulate in hosts with AML, confirming T cell deletion as a mechanism of potent peripheral tolerance in AML. Deletional T cell tolerance in this model could be reversed following administration of an agonistic anti-CD40 antibody to systemically activate host DCs, which not only significantly enhanced antigen-specific T cell responses in mice harboring AML cells systemically, but also improved their survival. These findings are important not only because we have identified a unique mechanism of immune evasion in AML, but also because they demonstrate a sharp contrast between how the immune system recognizes and is regulated by solid and hematological malignancies.

## Conclusions

Based on the discussion above, it should be clear that the tumor environment is well-equipped to subvert the host immune response generated against it. An emerging consensus is that future immunotherapies are likely to benefit from efforts to thwart tumor immune evasion. The evasion mechanisms employed by AML have only recently begun to be clarified, generating the next layer of fundamental questions for further investigation. For example, 1) What are the relative contributions of individual immune escape pathways in AML patients? 2) Does AML utilize immune evasion mechanisms that are yet to be discovered? 3) Do specific subtypes of AML differentially employ unique combinations of immune escape mechanisms? 4) How can strategies to reverse immune evasion be effectively coupled with standard immunotherapy approaches? 5) At what stage in the disease process should immune-based therapies (including strategies aimed at the reversal of immune evasion pathways) be delivered in order to maximize efficacy?

In this review, we have discussed the major immune evasion mechanisms with particular relevance to AML. Because AML is a systemic disease at its inception, the environment it creates is likely dissimilar in many aspects from anatomically-localized solid malignancies. Therefore, it seems reasonable to speculate that the immune evasion mechanisms employed in AML may differ from those in solid tumors. On the other hand, there is clear overlap in several immune escape pathways promoted by AML and solid tumors, such as expansion of Tregs, expression of negative regulatory receptors, production of IDO and lack of T cell costimulation. Truly unique immune tolerance mechanisms employed in AML have not been described, although our laboratory has recently characterized a deletional T cell tolerance mechanism in AML that that does not appear to be active in solid cancer models [102]. Here, T cells specific for leukemia-derived antigens were deleted, likely through interactions with immature host dendritic cells which cross-presented leukemia antigens in a context unfavorable to T cell activation, resulting in abortive T cell proliferation and dysfunctional cytokine production. It is reasonable to predict that additional mechanisms of T cell tolerance in AML will continue to be uncovered. Additionally, as distinct AML subtypes are further characterized in patients (i.e. core binding factor, FLT-3-mutated and Ras-mutated AML), it will be interesting to compare the immune escape pathways employed in these cohorts. If genetically similar AMLs reliably are found to exploit a common network of immune evasion mechanisms, it would provide strong rationale for developing clinical trials testing the reversal of specific immune evasion pathways in groups of patients who would be more likely to benefit.

Current immunotherapy strategies, such as vaccination and adoptive T cell transfer have historically not been beneficial for a majority of patients. However, animal models in which vaccine or adoptive T cell therapy have been combined with approaches to reverse immune evasion have yielded quite promising results. As discussed above, there are now several ongoing studies testing such combinations in cancer patients. In AML, our institution is coupling a WT1 peptide-based vaccine with Treg depletion (using an anti-human CD25 antibody), and other groups are exploring similar approaches (NCT01096602; NTC01513109). While the possible combinations to be explored are nearly endless, it is anticipated that as effective approaches to reverse immune evasion in leukemia patients are identified, they will be prioritized for study with standard immunotherapy.

Timing is a critical factor to consider when delivering immunotherapy in cancer, and AML is likely no exception. Clear evidence from animal models suggests that immunotherapy is less effective when administered to hosts with high tumor burden because of the well-established immune suppressive networks in larger tumors. In AML for example, patients with large numbers of marrow and/or peripheral blood blasts are certainly less likely to benefit from immunotherapy than those who have achieved a complete response. Because standard chemotherapy is effective at inducing complete remission in most AML patients (many of whom ultimately will relapse), there is reason to speculate that immunotherapy will provide the greatest benefit to patients in a minimal residual disease (MRD) state.

In conclusion, immune evasion in AML represents a major hurdle to the delivery of effective immunotherapy. With the recent characterization of several putative immune escape mechanisms in AML, opportunities to inhibit these pathways are becoming available. Emerging data from patients with solid malignancies suggests that circumventing these immunosuppressive pathways can lead to impressive objective clinical responses. However, there is much that remains to be explored both in the laboratory and in patients. In the future, we envision that targeting the reversal of immune evasion in combination with more classical immunotherapies, preferably delivered in the MRD state, may lead to the eventual incorporation of effective immune-based therapies into current treatment protocols to reduce the risk of AML relapse and improve overall outcomes for patients.

## Abbreviations

AML: Acute myeloid leukemia; WT1: Wilm’s tumor 1; TAA: Tumor-associated antigen; TSA: Tumor-specific antigen; Treg: Regulatory T cell; MDSC: Myeloid-derived suppressor cell; TIL: Tumor infiltrating lymphocyte; MHC: Major histocompatibility complex; HLA: Human leukocyte antigen; IFNγ: Interferon gamma; CTLA: 4 cytotoxic T lymphocyte associated antigen-4; PD-1: Programmed death-1; PD-L1: Programmed death-1 ligand; LAG-3: Lymphocyte activation gene −3; TIM-3: T cell immunoglobulin domain and mucin domain-3; Gal-9: Galectin-9; SHP: Src-homology domain-containing phosphatase; PI3K: Phosphatidylinositide-3 kinase; HIV: Human immunodeficiency virus; FoxP3: Forkhead box P3; IL-2: Interleukin-2; TGFβ: Transforming growth factor beta; IDO: Indolamine 2,3-dioxygenase; 1-MT: 1-methyltryptophan; MRD: Minimal residual disease.

## Competing interests

The authors declare that they have no competing interests.

## Authors’ contributions

RT and JK reviewed relevant literature and contributed equally to the drafting of the manuscript. Both authors’ read and approved the final manuscript.
